# Modification of Hot-Wire Anemometers Frequency Bandwidth Measurement Method

**DOI:** 10.3390/s20061595

**Published:** 2020-03-13

**Authors:** Paweł Ligęza

**Affiliations:** Strata Mechanics Research Institute of the Polish Academy of Sciences, 27, Reymonta str. 30-059 Cracow, Poland; ligeza@img-pan.krakow.pl

**Keywords:** hot-wire anemometer, frequency bandwidth, square-wave test, sine-wave test, constant-bandwidth anemometer

## Abstract

In measurements of fast-changing flows, one of the key issues is knowledge of the anemometer frequency bandwidth. In such measurements, the measurement technique often used is hot-wire anemometry. The determination and optimization of the measurement bandwidth of the hot-wire system is very important for the quality of the measurements carried out. One of the methods used is square-wave or sine-wave electrical testing. The article proposes modification of this method, which involves applying an electrical test signal wirelessly directly to the sensor, using transformer inductive coupling. This modification may in some cases be beneficial and find application in selected metrological problems. The article describes the modified method and its example application.

## 1. Introduction

Hot-wire anemometers (HWA), or anemometers with other heated measuring elements, are mainly intended for measuring velocities in fast-changing flows [1]. The most commonly used measuring system here is a constant-temperature anemometer, providing a wide transmission bandwidth of velocity fluctuations. This bandwidth can reach hundreds of kilohertz, and in special systems even one megahertz or more [2,3,4,5].

The use of a hot-wire anemometer in a measuring problem [6,7,8] requires the selection and optimization of the sensor and measuring system. Many factors related to metrological requirements, such as the tested measuring area, medium properties, flow structure, range of measured velocities and frequency spectrum of velocity fluctuations, should be taken into account here [9].

In the measurement of fast-changing flows in particular, the determination and optimization of the measurement bandwidth of the measuring system is important for the quality of the measurements carried out. Due to the difficulty of forming a standard fast-changing flow with strictly set and controlled parameters, determining the bandwidth of the hot-wire anemometer is not a trivial issue. Therefore, a number of methods have been developed for measuring the anemometer bandwidth. These methods can be divided into four main groups:

### 1.1. Measurement of the Frequency Bandwidth in a Reference Flow with Set Parameters

To create such a flow, rotating discs or rollers with holes modulating the flow velocity are used. Obstacles generating vortices, such as cylinders or blades placed in the flow, are also used. However, the possibility of producing a fast-changing flow with set and controlled parameters is very limited. Parameters of such a flow can be estimated on the basis of the phenomenon model or by making measurements with reference instruments with appropriate parameters, if such instruments are available [1].

### 1.2. Measurement of the Frequency Bandwidth Based on a Mathematical Model of the Measurement System

The development of a mathematical model of the anemometric measurement system and determination of its parameters allows conducting computer simulation tests. In simulation tests, we can model the flow of any set parameters and determine the response of the measuring system. In such tests, the anemometer frequency bandwidth can be determined and optimized [4,10,11].

### 1.3. Measurement of the Frequency Bandwidth by Physical Impact on the Sensor

Hot-wire anemometers do not respond only to a change in flow velocity. They are also sensitive to changes in other physical parameters, such as temperature, pressure, and composition of the medium or, for example, radiation. In particular, this method uses heat transfer to the sensor via a modulated laser beam or other radiation source. This phenomenon can be used indirectly to estimate the anemometer bandwidth. However, the difference in impact of velocity changes and other physical quantities should be considered. This method allows assessment of the anemometer bandwidth to a limited extent and requires appropriate equipment [12].

### 1.4. Measurement of the Frequency Bandwidth by a Test Electrical Signal

This method consists in providing a voltage test signal to the electronic system of the anemometer. Based on the response of the anemometric system to this signal, the frequency bandwidth is determined. Normally, a square or sinusoidal test signal is used. Due to the ease of implementation and the developed measurement methodology, this method is widely used and considered standard [1,4,13]. The electrical square-wave test method has been used in many commercial anemometers from companies such as Dantec (DISA) and TSI. However, it should always be taken into account that this is not a reference method and the results obtained are correct when the assumptions of this method are met with sufficient accuracy.

The article proposes modification of this method, which involves applying an electrical test signal wirelessly directly to the sensor, using transformer inductive coupling. This modification may in some cases be beneficial and find application in selected metrological problems. In particular, the method can be used in devices where it is not possible to connect the test signal by wire, or such a connection could disturb the operation of the device. The article further describes the method and example application.

## 2. Modification of the Frequency Bandwidth Measurement Method

The standard method of measuring the bandwidth of a hot-wire constant-temperature anemometer is to supply a voltage test signal to the electronic system of the anemometer. This signal is usually supplied as an additional unbalance (offset) voltage of the amplifier working in the feedback loop of the constant-temperature anemometer. Periodic changes of this voltage cause a temporary deviation of the system from equilibrium, and then the reaction of the system striving for equilibrium by a slight change in the temperature of the sensor. Based on the system response to this test excitation, the anemometer frequency bandwidth is determined. An appropriate methodology based on theoretical model research and experiments has been developed here [1,4,13]. Typically, square or sinusoidal electric voltage signals are used. For a square signal, the frequency bandwidth is determined on the basis of the shape and time of the response of the anemometric system to the excitation. Analysis of the frequency spectrum of the response signal may also be used [13]. The square-wave test methodology moreover allows shape assessment and optimization of the anemometer frequency bandwidth. Using a sinusoidal signal, the anemometer response to an increasing frequency signal is tested. The anemometer response amplitude is initially flat, and then increases linearly with a slope of 20 dB per decade. With a further increase in frequency, the response amplitude deviates from this straight line and begins to decrease. The bandwidth of the anemometer determines the frequency for which the deviation from the straight line is 3 dB. For the sinusoidal test, the methodology and the required apparatus are more complex compared to the square test. Therefore, the sinusoidal test is used less often. In the described methods, the test signal is supplied to the electronic system by wire. Often, the instruments are equipped with an internal test generator or an appropriate socket to supply a test signal from an external generator.

The author proposes a modification of this method, consisting in providing an electrical test signal wirelessly, directly to the sensor. Wireless connection of the test signal directly to the sensor is a new, original concept. This idea was based on the observation that anemometric systems, due to the kind of sensor, the high gain used and the wide frequency bandwidth, are very sensitive to external electromagnetic interference. It was decided to transform this disadvantage into a new concept of anemometer testing. The sensor of the hot-wire anemometer from an electrical point of view is an induction loop. It is therefore possible to supply an electrical test signal directly to the sensor via inductive coupling. The signal from the test generator supplies a miniature coil located near the sensor and inductively coupled to it. The test signal causes a current flow in the sensor circuit and an output signal response. The measurement methodology is similar to that used in the test with wire coupling. With proper selection of the signal source and the coupling coil parameters, it is possible to obtain a square, sinusoidal or other test signal of selected shape. The proposed modification is not a new method; however, in some applications, it may have beneficial properties, such as:

- It can be used in instruments without an internal frequency test, without interfering with the electronic system;
- It can be used in integrated measuring systems, MEMS and NEMS systems, and other systems without the possibility of a wired input of the test signal;
- Elimination of the influence of electronic components of the testing system on the properties of the electronic anemometric system (the wired connection of the test system introduces additional resistances, capacities and inductances to the electronic anemometer system that affect static and dynamic parameters);
- Supplying the test signal wirelessly, directly to the sensor;
- The possibility of testing multi-fiber sensors [14,15] with a common test signal;
- The possibility of implementing a test bench for multiple anemometers with the same wireless test signal.

**Figure 1:**
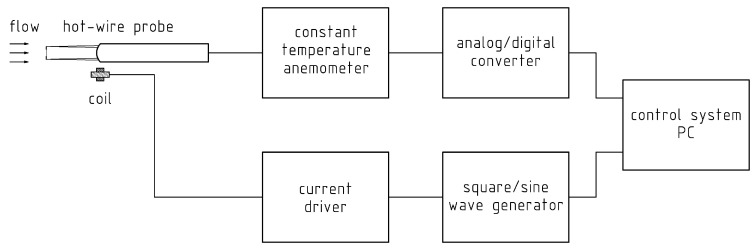
Schematic of a modified system for determining the frequency bandwidth of a constant-temperature anemometer.

The scheme of the modified system for determining the frequency bandwidth of a constant-temperature anemometer is shown in Figure 1.

The tested anemometer consists of a hot-wire sensor connected to an electronic constant-temperature system. The output signal from the anemometer through the analog–digital converter is fed to the measurement control system (PC). This system also controls a generator that creates a test signal. The signal from the generator through a current amplifier supplies the induction coil located near the hot-wire sensor. It is important to select the position and parameters of the coil to ensure permanent inductive coil-sensor coupling and the appropriate frequency bandwidth of this coupling. The hot-wire sensor was placed in the reference flow, which allowed determination of the frequency bandwidth of the anemometric system at different velocities. The measurement methodology is almost the same as for standard tests with a signal connected directly by wire to the anemometer electronic system.

## 3. Application of the Measuring Method

**Figure 2:**
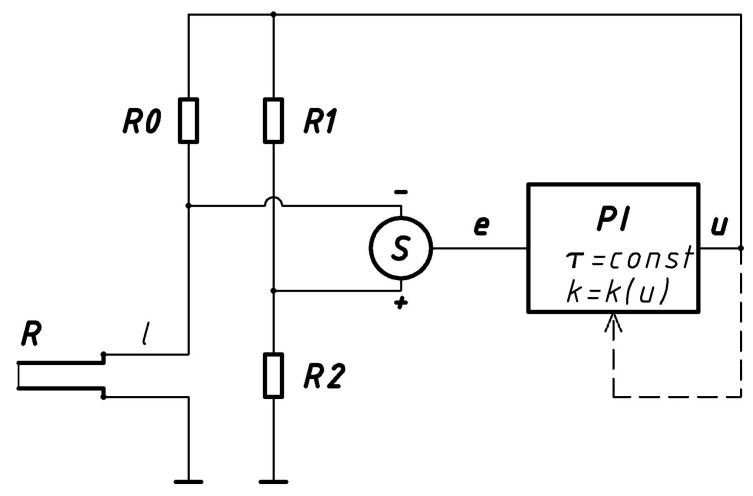
A simplified diagram of the constant-temperature constant-bandwidth anemometer.

Experimental studies were carried out to verify the presented idea. The modified method was used to determine and optimize the frequency bandwidth of a special instrument, a constant-temperature constant-bandwidth anemometer [16,17,18]. A simplified diagram of the constant-temperature constant-bandwidth anemometer is shown in Figure 2.

This circuit is an extended version of the standard bridge constant-temperature anemometer. The hot-wire sensor, *R*, is connected via a cable, *l*, to the Wheatstone bridge. The signal from the diagonal of the bridge is converted into an error signal, *e*, in the differential node, *S*. The error signal is fed to the proportional-integral controller, *PI*. The controller’s task is to supply the bridge in the feedback loop with such a voltage, *u*, as to balance the bridge by heating the sensor, *R*. The dynamic properties of the system depend mainly on the parameters of the sensor, bridge and controller. With constant controller parameters, the anemometer frequency bandwidth is a function of flow velocity. In high-amplitude, fast-changing and pulse flows this can be a serious source of dynamic errors. To eliminate these errors, a second feedback loop was introduced into the system. The constant-bandwidth anemometer is a type of constant-temperature anemometer in which the controller gain, *k*, is automatically controlled so as to obtain as constant as possible a frequency bandwidth over the whole velocity range. The controller time constant, *τ*, has a smaller effect on the frequency bandwidth, so it does not have to be changed. Since the electronic circuit of this anemometer has two feedback loops, a conventional frequency test may disrupt the operation of the system, so a wireless test was used. Constant-bandwidth anemometer tests were carried out with a tungsten fiber 5 micrometers in diameter and a length of 2 mm filament sensor. The sensor resistance at ambient temperature was about 5 ohm. *R0* was 10 ohm, and *R1* was 100 ohm. Resistance *R2* was chosen for obtaining the value of overheat ratio 1.8. Cable l type RG58 with a length of 1.5 m was used. 

**Figure 3:**
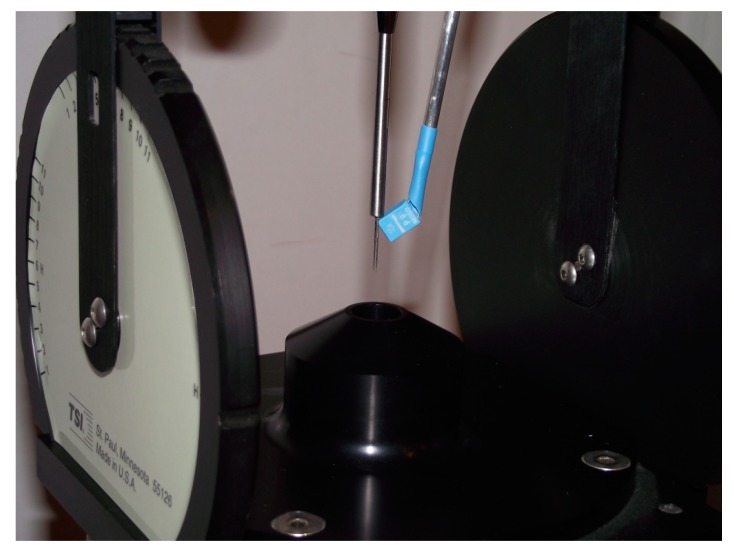
The research stand for the tests: nozzle, hot-wire sensor and coil.

The anemometric system was tested in two modes of operation, in the constant-bandwidth mode with a variable range between 220 and 1300 gain *k* of the controller, and for comparison with the constant bandwidth mode switched off, i.e., with constant gain *k* = 220. The time constant, *τ*, of the controller in both cases was 150 microseconds. The research stand for the tests is shown in Figure 3.

The air flow in the range up to 50 m/s was generated by the TSI calibration system model 1128, using a 14 mm nozzle. In the preliminary tests, a miniature induction element Neosid 10 k was used as the coil. The test signal was generated and recorded by means of an oscilloscope with generator Rohde & Schwarz RTM3K-24. The coil was supplied with a maximum voltage amplitude of 10 V by a simple current driver.

**Figure 4:**
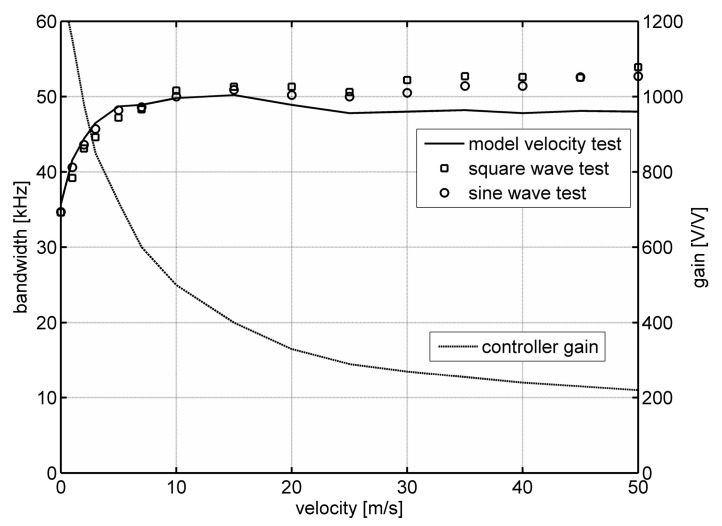
The measured dependence of the bandwidth of a constant-temperature constant-bandwidth anemometer on flow velocity.

Figure 4 shows the dependence of the bandwidth of a constant-temperature constant-bandwidth anemometer on flow velocity. Figure 4 shows the results obtained using square and sinusoidal tests. The solid line marks the results obtained in the numerical modeling process using the methodology and model from [16,17,18]. Figure 4 also presents changes in the constant-temperature controller gain, *k*, allowing for the implementation of the constant-bandwidth operation.

**Figure 5:**
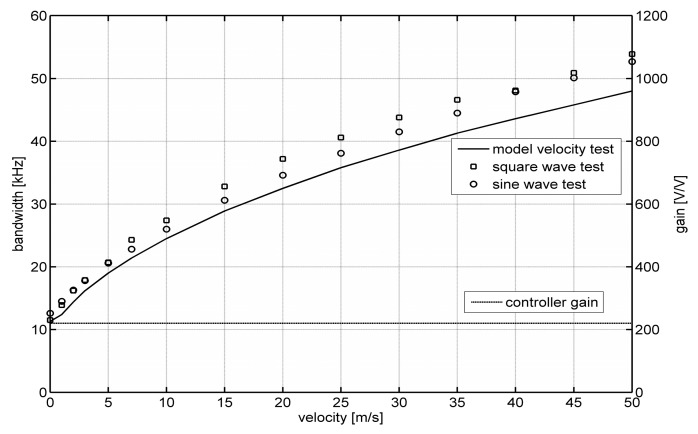
Dependence of the bandwidth of a constant-temperature anemometer on flow velocity when the constant-bandwidth function is off.

For comparison, Figure 5 shows the determined dependence of the bandwidth of a constant-temperature anemometer on flow velocity when the constant-bandwidth function is off. As before, the results of the square and sinusoidal tests are presented as well as those obtained in the modeling process. The gain, *k*, of the constant-temperature controller is in this case constant, optimally selected for the highest velocity.

## 4. Conclusions

Preliminary verification of the described measurement method allows us to state that the method can be used to determine the frequency bandwidth of hot-wire anemometers. The frequency bandwidth determined by square and sinusoidal tests is similar. The results are also consistent with the results obtained by standard methods [16,17,18]. The frequency bandwidth determined in model tests has a similar course, however the obtained values are on average about 5% lower. This results from both the limited accuracy of the adopted model and the uncertainty in determining its parameters [9,11,16,18]. The conducted tests also confirm the unique properties of the constant-temperature constant-bandwidth anemometer. The frequency bandwidth determined for the presented case is practically flat above the velocity of several meters per second.

The presented modification of the method for determining the bandwidth of hot-wire anemometers certainly will not replace standard methods but may be beneficial in some cases mentioned in the article. It is also possible to use the proposed solution in testing MEMS and NEMS measurement systems [19], and other integrated instruments and systems [20,21,22].
